# Interacting with wildlife tourism increases activity of white sharks

**DOI:** 10.1093/conphys/coy019

**Published:** 2018-06-06

**Authors:** Charlie Huveneers, Yuuki Y Watanabe, Nicholas L Payne, Jayson M Semmens

**Affiliations:** 1 College of Science and Engineering, Flinders University, Bedford Park, Adelaide, South Australia 5165, Australia; 2 National Institute of Polar Research, Tachikawa, Tokyo 190-8518, Japan; 3 SOKENDAI (The Graduate University for Advanced Studies), Tachikawa, Tokyo 190-8518, Japan; 4 University of Roehampton, London SW154JD, UK; 5 Fisheries and Aquaculture Centre, Institute for Marine and Antarctic Studies, University of Tasmania, Taroona, Tasmania 7053, Australia

**Keywords:** accelerometry, *Carcharodon carcharias*, ecotourism, energy budget, metabolic rate

## Abstract

Anthropogenic activities are dramatically changing marine ecosystems. Wildlife tourism is one of the fastest growing sectors of the tourism industry and has the potential to modify the natural environment and behaviour of the species it targets. Here, we used a novel method to assess the effects of wildlife tourism on the activity of white sharks (*Carcharodon carcharias*). High frequency three-axis acceleration loggers were deployed on ten white sharks for a total of ~9 days. A combination of multivariate and univariate analysis revealed that the increased number of strong accelerations and vertical movements when sharks are interacting with cage-diving operators result in an overall dynamic body acceleration (ODBA) ~61% higher compared with other times when sharks are present in the area where cage-diving occurs. Since ODBA is considered a proxy of metabolic rate, interacting with cage-divers is probably more costly than are normal behaviours of white sharks at the Neptune Islands. However, the overall impact of cage-diving might be small if interactions with individual sharks are infrequent. This study suggests wildlife tourism changes the instantaneous activity levels of white sharks, and calls for an understanding of the frequency of shark-tourism interactions to appreciate the net impact of ecotourism on this species’ fitness.

## Teaser

With wildlife tourism rapidly expanding, it is critical to understand its effects on the species it targets. Accelerometer loggers show that white shark activity increases when they are interacting with cage-diving operators. The study highlights the importance of considering bioenergetics when evaluating the effects of wildlife tourism on wildlife.

## Introduction

Understanding how species respond to human-induced changes has become an important research pursuit (Sih *et al.*, 2011; Tuomainen and Candolin, 2011; Robbins *et al.*, 2017). Wildlife tourism is a human activity that can modify the natural environment and behaviour of the species it targets through habitat modification or food provisioning (Green and Higginbottom, 2001; Orams, 2002). In recent decades, wildlife tourism has been rapidly expanding and has become one of the fastest growing sectors of the tourism industry (Scheyvens, 1999; Wearing and Neil, 2009).

Research on the effects of tourism on elasmobranchs is on the rise, with previous studies investigating impacts of provisioning on elasmobranch physiology (e.g. Semeniuk *et al.*, 2007; Barnett *et al.*, 2016), changes in seasonality, residency or abundance (e.g. Meyer et al., 2009; Bruce and Bradford, 2013; Brunnschweiler and Barnett, 2013), changes in space use (e.g. Corcoran *et al.*, 2013; Huveneers *et al.*, 2013), changes in vertical activity (e.g. Fitzpatrick *et al.*, 2011; Huveneers *et al.*, 2013), and physical effects from divers (e.g. Smith *et al.*, 2010). Whether these changes lead to reduced fitness at the individual or population levels is mostly unknown and has been identified as requiring further investigation (Brena *et al.*, 2015; Gallagher *et al.*, 2015). The ability of wildlife tourism to affect individual fitness and survival has been documented in terrestrial (e.g. Orams, 2002), avian (e.g. Steven *et al.*, 2011), and aquatic species (e.g. Bejder *et al.*, 2006; Williams *et al.*, 2006), and is reviewed in Green and Giese (2004), but similar studies on elasmobranchs are limited.

The white shark (*Carcharodon carcharias*) is a large marine apex predator with a global distribution, occurring in temperate, sub-tropical and tropical waters (Klimley and Ainley, 1996; Domeier, 2012). The elusive nature, size and involvement of the species in fatal shark–human interactions has led white sharks to be considered a charismatic species that is often targeted by ecotourism (Apps *et al.*, 2016, 2017; Huveneers *et al.*, 2017). Commercial white shark cage-diving uses olfactory, visual, or auditory attractants several hours per day every day to attract sharks within close proximity of the cages and to provide good viewing opportunities for divers. In contrast to many other shark-related tourism (e.g. Brunnschweiler and Barnett, 2013), provisioning is limited as operators are not permitted to intentionally feed white sharks. However, they can occasionally consume baits when operators are unable to detect rapidly approaching sharks. Ecotourism opportunities are now available in five countries (Australia, South Africa, the USA, Mexico and New Zealand), with up to seven different businesses operating simultaneously at one site and some cage-diving operators hosting up to three expeditions per day. This has led to concerns in some jurisdictions about the potential for cage-diving activities to alter the behaviour of white sharks. These concerns have been supported by previous studies which have found that the cage-diving industry can change the fine-scale 3D spatial distribution, rate of movement, residency and temporal distribution of white sharks (Bruce and Bradford, 2013; Huveneers *et al.*, 2013). However, it is unknown whether these changes have any long-term effect on physiology, energy balance, or fitness and ultimately population viability, as changes in behaviour do not necessarily indicate changes in health or fitness (Beale and Monaghan, 2004; Gill *et al.*, 2001). Only one study has previously investigated ecotourism-related changes in energy expenditure in sharks (Barnett *et al.*, 2016); a study that showed provisioning whitetip reef sharks (*Triaenodon obesus*) for tourism increases their daily energy expenditure by elevating activity levels during periods when they normally rest (Barnett *et al.*, 2016). The metabolic rate of whitetip reef sharks increased by 6.37%, which is comparable to half the proportion of energy similar shark species contribute to growth.

Advances in tagging technology now allows researchers to assess changes in energy expenditure using proxies such as tailbeat frequency or activity levels (Cooke *et al.*, 2016). The present study used three-axis acceleration loggers to compare activity of white sharks and examine a range of behaviour and performance metrics in relation to the operations of a cage-diving industry at the Neptune Islands (South Australia). Specifically, we hypothesised that the activity of white sharks would increase when cage-diving vessels were present at the Neptune Islands and increase further when in close proximity to the cage-diving vessels. The findings from this study provide critical information to assess the potential effect of wildlife tourism targeting sharks, and ultimately improve our understanding of behavioural responses to anthropogenic influences.

## Methods

### Study site and white shark cage-diving industry

**Figure 1:**
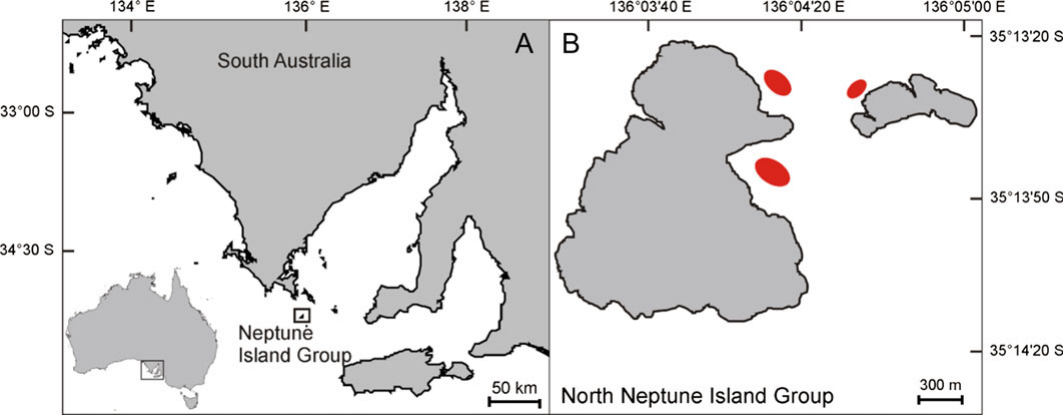
Location of the Neptune Islands Group Marine Park and areas where cage-diving operators typically anchor at the North Neptune Islands Group (red ellipses).

The Neptune Island group is located near the approach to Spencer Gulf, about 30 km from the South Australian mainland (Fig. 1). While the waters surrounding the South and North Neptune Island groups are open to cage-diving operations, the North Neptune Islands group (35°149 S; 136°049 E) is most frequently used by the current operators. Two cage-diving vessels use a near-constant odour corridor of berley (or chum) during daylight hours, comprising a mix of minced southern bluefin tuna (*Thunnus maccoyii*) products to attract sharks present in the area to the vessels. Tethered baits of tuna sections or gills and entrails of up to several kilograms are also used to improve client experience by keeping sharks within visual range of divers in the cage. The third operator does not use berley or tethered bait but uses sound transmitted from an underwater speaker to attract sharks.

### Equipment and deployment

White sharks were equipped with an ‘accelerometer package’ that included several devices to record the activity of white sharks and ensure the recovery of the device (Watanabe *et al.*, 2004; Watanabe and Sato, 2008) during August–September 2014, October–November 2015 and January 2016. The accelerometer package included: (1) a multi-sensor data logger (W1000-PD3GT, 21-mm diameter, 115-mm length and 60 g; Little Leonardo) that recorded relative swim speed as the number of rotations of a propeller, depth, temperature (all at 1 Hz frequency) and three-axis acceleration (at 16 or 32 Hz frequency); (2) a very high frequency (VHF) radio transmitter (Advanced Telemetry Systems) and (3) an Argos transmitter (SPOT; Wildlife Computers).

**Figure 2:**
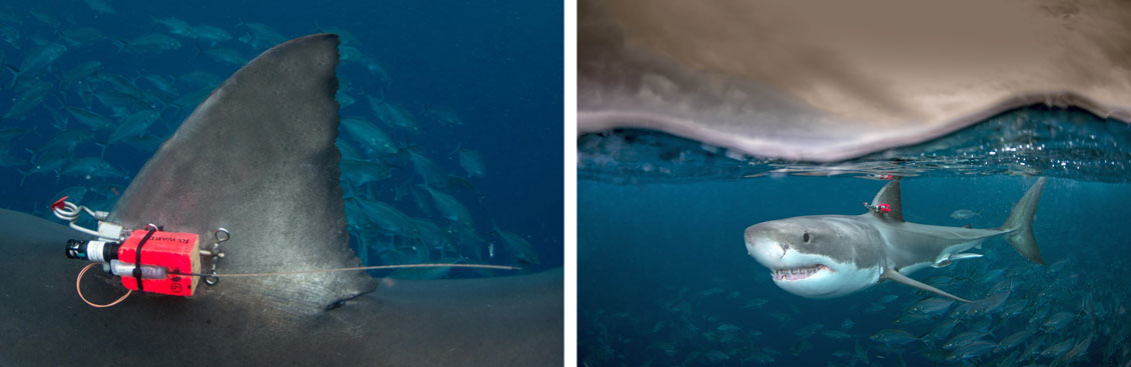
Example of an accelerometer package deployed on a white shark (*Carcharodon carcharias*).

A plastic cable connected to a time-scheduled release mechanism (Timer RT-5; Little Leonardo) bound the package to a fin-clamp (Chapple *et al.*, 2015). Once the release mechanism had been activated after a 1–2-day free-swimming period, the plastic cable was severed by an electric charge from the battery of the device, and the whole buoyant package was released from the shark and floated to the surface. The package was located by using VHF and Argos signals and recovered by boat. Accelerometer packages were clamped, using a deployment pole (Chapple *et al.*, 2015), on to the first dorsal fin of sharks that were attracted to the vessel (Fig. 2). Accelerometer packages were positioned at the base of the fin on each shark to minimise variations due to differing accelerometer placement. The clamp had a corrodible link incorporated in it to allow it to break off and release from the dorsal fin after ~1 week.

### Experimental design

For each shark, the total deployment period was divided into shorter periods, which were assigned one of five contexts based on time of the day, the presence/absence of the shark and cage-diving operators, and the behaviour of the shark (Table 1). The presence of sharks at the Neptune Islands was determined based on swimming depth and validated by the pop-up location of the accelerometer package (e.g. the package sometimes popped-up at the cage-diving site and in some cases popped-up 10 km away from the Neptune Islands). The area where cage-diving takes place is up to ~40 m deep, with the depths around the Neptune Islands rapidly increasing and reaching more than 70 m within 500 m of the Islands. Sharks, therefore, need to leave the vicinity of the Neptune Islands and area of cage-diving operation to reach depths greater than ~70 m. Sharks were considered away from the Neptune Islands when maximum depths were > 70 m for longer than 30 min. Sharks were considered present at the Neptune Islands in all other contexts. It is theoretically possible that sharks left the vicinity of the Neptune Islands but remained within the top 70 m of the water column. Previous satellite studies have, however, shown that white sharks frequently reach the seafloor (Bruce *et al.*, 2006; Sims *et al.*, 2012), supporting the use of maximum depth to identify when sharks left the Neptune Islands.

**Table 1:** Description of the contexts used to categorise the activity of each shark

Context code	Context description
Interaction	At least one cage-diving operator present and shark actively engaging with one of the operators by attempting to consume teaser bait or swimming around the cage or vessel. A shark was considered to be interacting with cage-diving operators if within 20 m of the bait or cage-diving vessel and with excursions away from the vessel < 10 min. If the shark was not sighted for > 10 min, it was then considered to be in the ‘Operator present’ context (see below) until sighted again within 20 m of the bait or vessel. The presence of the shark was determined by cage-diving operators keeping a record of interactions with sharks that carried the accelerometer package. The sharks could be easily identified as the accelerometer package was clearly visible on the first dorsal fin. In cases when an accelerometer package was deployed on several sharks concurrently, sharks could be differentiated from differences in size, scars and pigmentation, Nasby-Lucas and Domeier (2012).
Operator present	At least one cage-diving operator present and shark present based on maximum swimming depth ≤70 m.
Operator absent	Shark at the Neptune Islands based on maximum swimming depth ≤70 m but no cage-diving operators present.
Night	Period between sunset and sunrise. Cage-diving does not take place at night.
Outside	Shark away from the Neptune Islands as determined by swimming depth (maximum swimming depths > 70 m for longer than 30 min).

### Data processing

Swim speeds (m/s) were estimated from logger propeller rotation values, using a relationship between rotation and speed determined in a flow-tank. Attachment angle (i.e. angle between the animal’s body axis and the logger’s longitudinal axis estimated following Kawatsu *et al.* (2009)) was accounted for in the estimation of swim speed from propeller rotations using trigonometry, and validated in a flow-tank. Average overall dynamic body acceleration (ODBA) was calculated by removing the static contribution of gravity from acceleration data using a high-pass filter, and then summing the absolute values of acceleration from all three axes. ODBA is often used as a measure of overall animal activity level and instantaneous rate of energy expenditure (Wilson *et al.* 2006). The use of body acceleration as a proxy for metabolic rate or energy expenditure is based on the principle that animal movement results directly from muscle contraction, which is catalysed by adenosine triphosphate (ATP) hydrolysis and thus requires oxygen (Wilson *et al.*, 2006; Gleiss *et al.*, 2011). Tailbeat frequency was estimated from lateral acceleration by a Fast Fourier Transformation using Ethographer (Sakamoto *et al.*, 2009). Burst events were defined as a period during which ODBA was greater than 0.5 g based on visual inspection of ODBA and swim speed to identify sudden and dramatic increase that clearly represented an increased activity above steady-state swimming. The number of burst events were calculated and presented as the number of burst events per hour. Ascents were defined using depth differences between consecutive records. Depth difference was calculated by taking the central difference of the depth over 1 s intervals after which the trace was smoothed. Ascent phases were defined as periods when depth difference was more than −0.2 m/s. Each ascent phase represented a single event and enabled the number of ascents per minute to be calculated.

### Statistical analysis

Multivariate analyses were conducted using PRIMER v7 (Clarke and Gorley, 2006) and the PERMANOVA+ add-on package (Anderson *et al.*, 2008). A resemblance matrix was produced using Euclidean distance. Permutational multivariate analysis of variance (PERMANOVA) was used to test if shark activity, as measured via ODBA, swim speed, tailbeat frequency, and number of bursts and ascents, were different between contexts. Non-metric multidimensional scaling (nMDS) was used to visualise the data as an unconstrained ordination.

The influence of the activity metrics were further investigated through univariate analyses. Generalised linear mixed models (GLMM) were used to determine the effects of contexts on each of these influential metrics, with Interaction as the base level and being compared against the other contexts. The inclusion of individual shark as a random effect enabled the analysis to account for the lack of independence in behaviour within each identified shark. The most appropriate statistical family, error distribution, and validity of the model were determined through an examination of the distribution of the response variable, a visual inspection of the residuals for the saturated models, and an ANOVA test between the fitted and residual values of the model. Modelling was undertaken using the ‘glmmPQL’ function of the MASS R package and accounting for serial autocorrelation. Model fitness was assessed based on R squared values following Edwards *et al.* (2008) extended to the GLMM using penalised quasi-likelihood estimation by Jaeger *et al.* (2017).

## Results

The accelerometer package was deployed on ten sharks (9 males, 1 female) between 2.9 and 4.3 m total length for a total of 211.5 h (Table 2). The packages were deployed for 30 min–39 h 40 min (mean ± standard error: 21 h 13 min ± 4 h 22 min). The swim speed of two sharks (shark 6 and 10) was excluded from the analysis because the deployment angle of the fin-clamp was too far from the horizontal to apply the correction factor. The number of tagged sharks did not allow for a test of the effect of size or sex. Not every shark collected data from all contexts, but all sharks interacted with cage-diving operators. Some sharks interacted with operators the entire daytime deployment period (e.g. Shark 1), while others were at the Neptune Islands when operators where present, but spent most of their time away from the operators (e.g. Shark 4 and 8). Shark 7, 9 and 10 spent some of the deployment period away from North Neptune Islands. Shark 7 swam from North Neptune Islands where the accelerometer package was deployed to South Neptune Islands, ~11 km away, where the shark was re-sighted with the accelerometer package. The accelerometer package of Shark 9 and 10 popped-up and was recovered ~10, and 14 km away from North Neptune Islands, respectively.

**Table 2:** Sex and length of white sharks (*Carcharodon carcharias*) on which the accelerometer package was deployed; the period during which the accelerometer package recorded data is indicated for each shark and Context.

Shark	Sex	Total length (m)	Deployed period (min)					
			Absent	Present	Interaction	Outside	Night	Total
1	Male	3.3			263		762	1025
2	Male	3.2			412		832	1244
3	Male	4.3		60	65			125
4	Male	4.3		639	232.5		1507.5	2379
5	Female	4.2	180	149	138		756	1223
6	Male	3.5			34			34
7	Male	3.8	82	97	53	566	636	1434
8	Male	2.9		293	17		505	815
9	Male	3.7			34	60	632	726
10	Male	3.5	386		10	658	1186	2240
		Total	648	1238	995.5	1284	6816.5	11 245

**Figure 3:**
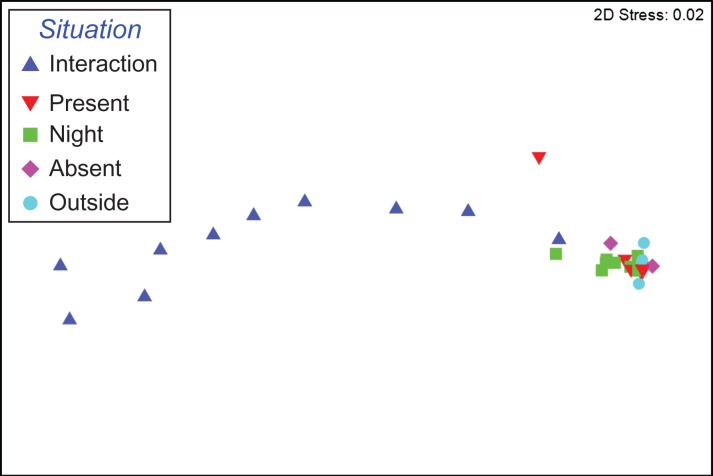
Non-metric multidimensional scaling (nMDS) plot based on Euclidean distances, showing (*N* = 29) contexts from 10 white sharks (*Carcharodon carcharias*). Contexts (symbols) that are more similar to one another are ordinated closer together. Stress = 0.02.

**Figure 4:**
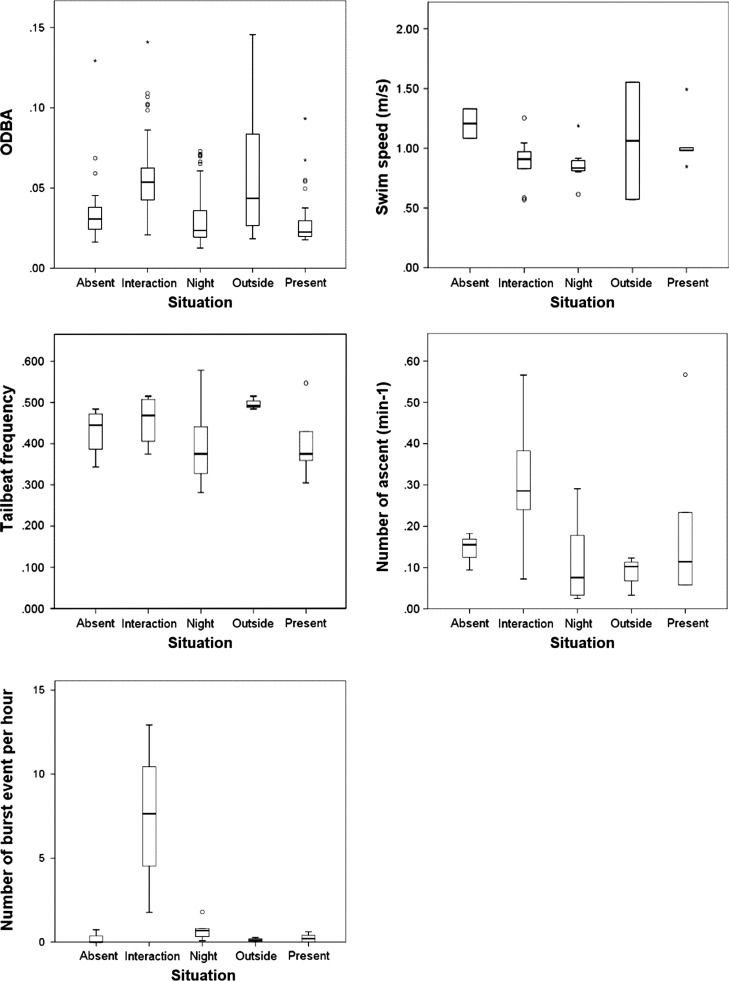
Plots showing the effects of different contexts on white shark activity metrics. Median values are indicated by the bold horizontal bar; the length of the box is the inter-quartile range; whiskers represents 1.5 inter-quartile range; circles are outliers; and asterisks are extreme values.

The activity of white sharks varied significantly between contexts (PERMANOVA: df = 4; MS = 105.05; Pseudo-*F* = 18.195; *P*(perm) = 0.0002), with Interaction being significantly different to all other contexts, but no other contexts being different to each other (Table 3). The nMDS shows that Interactions were separated from the rest of the contexts, while all other contexts clustered together (Fig. 3). The lack of clustering during Interactions is likely due to behavioural differences between individuals being greater than during other contexts. The GLMM results supported the multivariate analyses and showed that most activity metrics of white sharks during Interaction were different to the other contexts (Table 4; Supplementary Table S1; Fig. 4). Specifically, the number of burst events during Interactions was significantly higher than in other contexts. Average ODBA was significantly higher during interactions compared to all other contexts when sharks were at the Neptune Islands. The number of ascents significantly increased when interacting with the operators. However, swim speed was not significantly different across contexts (average swim speed = 0.94 ± 0.05 m/s; max swim speed = 3.93 ± 0.18 m/s), while tailbeat frequency during Interactions was only significantly different to periods when cage-diving operators were Present (Table 4; Fig. 4). The combination of the multivariate and univariate analyses suggests that the difference in behaviour during Interaction was mostly driven by the increased number of burst events and ascents, resulting in a higher ODBA compared to other times when sharks are present in the area.

**Table 3:** Summary of pairwise test between contexts.; *P*(perm) values in bold show values < 0.05

Contexts	*t*-Value	*P*(perm)
Interaction, Present	4.386	**0.002**
Interaction, Night	5.736	**<0.001**
Interaction, Absent	3.744	**0.001**
Interaction, Outside	3.789	**0.005**
Present, Night	0.929	0.427
Present, Absent	0.758	0.682
Present, Outside	0.912	0.480
Night, Absent	1.477	0.162
Night, Outside	1.724	0.069
Absent, Outside	0.427	0.799

**Table 4:** Summary of *P*-values from the generalised linear mixed models (GLMM) with indication of family and link function selected through an examination of the distribution of the response variable, a visual inspection of the residuals for the saturated models, and an ANOVA test between the fitted and residual values of the model; Interaction is the base level against which all other contexts are compared; values in bold show *P*-value < 0.05; value in brackets in the ‘Activity metric’ column represents *R* squared value for GLMM computed using Jaeger *et al.*’s (2017) method.

Activity metric (*R*^2^)	Family (link function)	Interaction	Absent	Night	Outside	Present
ODBA (0.58)	Gamma (log)	Base level	**0.0166**	**<0.001**	0.8304	**0.0010**
Swim speed (0.12)	Gamma (sqrt)	Base level	0.2670	0.2761	0.5635	0.3254
Tailbeat frequency (0.13)	Quasi (inverse)	Base level	0.3382	**0.0421**	0.4963	0.2860
No of ascents (0.55)	Gamma (log)	Base level	**0.0324**	**<0.001**	**<0.001**	0.0991
No of bursts (0.84)	Quasipoisson (log)	Base level	**0.0012**	**<0.001**	**0.0053**	**<0.001**

## Discussion

Our study suggests that the activity of white sharks increases during interactions with cage-diving operators and that the cage-diving industry has the potential to affect the energy budget of white sharks. The difference in the activity of white sharks was related to the number of burst events which increased by 10- to 60-fold when sharks were in proximity of the cage-diving vessels compared with all other situations. The vertical distribution of the sharks also became more variable as shown by the increased number of ascents during interactions with the cage-diving vessels. Together, these results reflect an overall change in white shark behaviour during interaction with operators and an increase in ODBA. Such behavioural change has previously been observed off Gansbaai (South Africa), where white sharks associating with cage-diving vessels switch from patrolling to area restricted searching (Towner *et al.*, 2016), with the associated tortuous movements expected to lead to an increase in ODBA.

ODBA increased from ~0.034 when sharks were at the Neptune Islands (i.e. ‘Absent’ and Present contexts) and at ‘Night’ to ~0.055 during Interactions, representing a 61% increase. Considering that several studies have validated ODBA as a reliable proxy for metabolic rate (Wilson *et al.*, 2006; Gleiss et al., 2011; Lear et al., 2017), this increase in ODBA suggests that the cage-diving industry has the potential to influence white shark energy expenditure. ODBA was, however, also high when white sharks were away from the Neptune Islands (~0.062), likely linked to sustained periods of fast swimming while travelling between foraging locations, as seen in other top predators (Block *et al.*, 2011).

While ODBA was significantly higher during Interactions, swim speed and tailbeat frequency were similar between contexts. The number of bursts and change in manoeuvring during Interactions might have not been accompanied by measurable changes in mean speed or tailbeat frequency. Tailbeat frequency was also based on dominant frequency from Fast Fourier Transformation, which is best estimated during regular swimming. Extensive turns and body movement such as during Interactions might affect the ability to identify dominant frequency. Swim speed was not estimated for two sharks because of the deployment angle of the accelerometer package, and there were a few instances when the swim speed stopped recording during strong acceleration. These might explain the discrepancy between swim speed, tailbeat frequency and ODBA, and result in ODBA being a better proxy for activity in this study than swim speed or tailbeat frequency.

Although interacting with the cage-diving industry increases ODBA while at the Neptune Islands, the amount of time that white sharks interact with operators is variable (Huveneers *et al.* 2013) and the presence of operators and berley does not by itself affect the activity of white sharks unless they are interacting with operators. A typical cage-diving day might, therefore, only result in a small overall increase in daily energy expenditure, as was suggested for the impact of provisioning on energy budgets of *T. obesus* (Barnett *et al.*, 2016). Further, the small increase in daily energy expenditure for *T. obesus* is for a species which typically spends a large portion of the day resting on the seabed (Barnett *et al.*, 2016), so perpetually active species such as white sharks could be expected to suffer smaller increases in provisioning-imposed energy expenditure. In addition, white sharks do not always come in close proximity to operators and can ignore them. For example, sharks can be within a cage-diving site, but not be sighted by operators (Delaney *et al.*, 2012) and can remain more than 200 m away from cage-diving operators throughout the day (Huveneers *et al.*, 2013). The amount of time sharks spend in close proximity to cage-diving operators is also highly variable between individuals (Huveneers *et al.*, 2013), and some sharks have been suggested to reduce their response to the olfactory and visual stimulus through time (Laroche, 2006). These show that a better understanding of the amount of time sharks spent in proximity to or interacting with cage-diving operators and its variation between individuals and through time is required to be able to assess the potential impact of cage-diving on the energy budget of white sharks.

Although sharks are enticed to the cage-diving vessels with baits, industry regulations do not allow operators to feed white sharks and strict limits on the amount of bait and berley are now in place in South Australia and at other white shark cage-diving locations (Bruce, 2015). Energy burden from the increased activity is, therefore, not rewarded by regular bait provisioning. Some baits can, however, be consumed when sharks approach the baits using high speed or stealth (Huveneers *et al.*, 2015). The baits used in SA are composed of gills and stomach lining of southern bluefin tuna and are not as energy-rich as white shark’s natural prey while at these sites (e.g. pinnipeds). Whether the infrequent consumption of these baits provide sufficient energy to compensate for the increased energy expenditure associated with sharks interacting with the operators would depend on the calorific value of these baits and the frequency of white sharks successfully feeding on the baits, both of which are currently unknown (Brunnschweiler *et al.*, 2017). Spending time interacting with cage-diving operators might also distract sharks from normal behaviours such as foraging on natural, energy-rich prey like pinnipeds. Combined, these suggest that the increased energy expenditure associated with cage-diving interactions might not be compensated for by either bait or natural prey consumption. One could, therefore, argue that white sharks should be able to feed on some bait to compensate for the energetic losses resulting from interacting with cage-diving operators. Bioenergetic models (e.g. Barnett *et al.*, 2016) would, however, be necessary to accurately assess the likely effect of cage-diving on white shark energy balance and whether such compensation is necessary or beneficial. Beyond the potential for short-term energy intake, other aspects of food provisioning (e.g. quality of food, potential for changes in foraging behaviour) would also need to be considered.

The white shark cage-diving industry in South Australia is managed using an adaptive management framework based on the residency of white sharks at the Neptune Islands, which is estimated annually (Huveneers and Lloyd, 2017). The current policy uses decision points in relation to white shark residency as indicators of the impacts of the cage-diving industry on the behaviour of white sharks. Under this policy, the number of days operators are allowed to be at the Neptune Islands and undertake cage-diving is modified according to changes in white shark residency (Smith and Page, 2015). While this policy has been successful at returning the increased residency of white sharks back to baseline levels of ~10 days per visit in 2001–03 (Huveneers and Lloyd, 2017), it focuses on a relatively coarse measure of the possible impact of the cage-diving industry. Wildlife tourism can have physiological and population-level impacts that might not be accounted for when only using coarse metrics such as residency, highlighting the importance of considering all aspects of disturbance when evaluating effects of human disturbance on wildlife (Christiansen and Lusseau, 2015).

The present study provides evidence of the effect of wildlife tourism on the activity of a marine apex predator and potential implications for its daily energy budget, and ultimately improves our understanding of behavioural responses to anthropogenic influences. Future research should quantify the amount of time white sharks interact with cage-diving operators and estimate its effect on white sharks in relation to their daily energy budget. Estimation and comparison of the energy obtained from natural prey vs. bait would also facilitate a better understanding of the effect of the cage-diving industry on the energy budget of white sharks (Brunnschweiler *et al.*, 2017). Such information will enable managers to go beyond the use of presence/absence of cage-diving vessels and sharks and account for the potential effect of wildlife tourism on the energy balance, fitness and ultimately population viability of this internationally threatened species.

## Supplementary Material

Supplementary Data
