# Supplementary material for: Interacting with wildlife tourism increases activity of white sharks
Source: Conserv Physiol. 2018 Jun 6;6(1):coy019. doi: 10.1093/conphys/coy019 (PMC5912080; doi:10.1093/conphys/coy019)
Supplement: Supplementary Data [file supplementarymaterial.docx]

**Supplementary material**

***Interacting with wildlife tourism increases activity levels of white sharks***

Charlie Huveneers, Yuuki Y. Watanabe, Nicholas L. Payne, Jayson M. Semmens

| Table S1. Summary of generalized linear mixed model (GLMM) outputs. DF = degrees of freedom | | | | | |
| --- | --- | --- | --- | --- | --- |
|  | **Value** | **Std Error** | **DF** | **t-value** | **p-value** |
| ***ODBA*** |  |  |  |  |  |
| Intercept | -2.938 | 0.116 | 15 | -25.31 | <0.001 |
| Absent | -0.372 | 0.138 | 15 | -2.70 | 0.0166 |
| Night | -0.605 | 0.094 | 15 | -6.42 | <0.001 |
| Outside | -0.030 | 0.139 | 15 | -0.22 | 0.8304 |
| Present | -0.457 | 0.113 | 15 | -4.06 | 0.001 |
| ***Swimming speed*** |  |  |  |  |  |
| Intercept | 0.934 | 0.043 | 12 | 21.87 | <0.001 |
| Absent | 0.044 | 0.038 | 12 | 1.16 | 0.267 |
| Night | -0.023 | 0.020 | 12 | -1.14 | 0.2761 |
| Outside | 0.020 | 0.033 | 12 | 0.59 | 0.5635 |
| Present | 0.025 | 0.024 | 12 | 1.03 | 0.3254 |
| ***Tailbeat*** |  |  |  |  |  |
| Intercept | 2.231 | 0.128 | 15 | 17.47 | <0.001 |
| Absent | 0.160 | 0.161 | 15 | 0.99 | 0.3382 |
| Night | 0.263 | 0.118 | 15 | 2.22 | 0.0421 |
| Outside | 0.093 | 0.134 | 15 | 0.70 | 0.4963 |
| Present | 0.157 | 0.142 | 15 | 1.11 | 0.286 |
| ***No of Ascent*** |  |  |  |  |  |
| Intercept | -1.256 | 0.227 | 15 | -5.53 | <0.001 |
| Absent | -0.858 | 0.364 | 15 | -2.36 | 0.0324 |
| Night | -1.156 | 0.251 | 15 | -4.61 | <0.001 |
| Outside | -1.638 | 0.365 | 15 | -4.48 | <0.001 |
| Present | -0.523 | 0.298 | 15 | -1.76 | 0.0991 |
| ***No of burst*** |  |  |  |  |  |
| Intercept | 1.976 | 0.154 | 15 | 12.85 | <0.001 |
| Absent | -3.562 | 0.891 | 15 | -4.00 | 0.0012 |
| Night | -2.360 | 0.335 | 15 | -7.04 | <0.001 |
| Outside | -4.013 | 1.232 | 15 | -3.26 | 0.0053 |
| Present | -3.450 | 0.690 | 15 | -5.00 | <0.001 |
